# Brücke zwischen Wissenschaft und Praxis im Öffentlichen Gesundheitsdienst: Erfahrungen aus dem Trainee-Rotationsprogramm *EvidenzÖGD*

**DOI:** 10.1007/s00103-025-04160-z

**Published:** 2025-12-01

**Authors:** Laura Arnold, Simon Bimczok, Nico Dragano, Simon Götz, Anke Kietzmann, Michael Schäfer, Hannah Schütt, Max Skorning, Franziska Vosseberg, Simone Weyers, Dagmar Starke, für das EvidenzÖGD Projektkonsortium, Laura Arnold, Simon Bimczok, Marlene Lakemann, Hannah Schütt, Dagmar Starke, Franziska Vosseberg, Nico Dragano, Delbar Dilmaghani, Annika Höhmann, Simon Götz, Simone Weyers, Ravina Ambalavanar, Anke Kietzmann, Andrea Melville-Drewes, Guido Schenuit, Trudpert Schoner, Max Skorning, Michael Schaefer

**Affiliations:** 1 Akademie für Öffentliches Gesundheitswesen, Düsseldorf, Nordrhein-Westfalen Deutschland; 2 Institut für Medizinische Soziologie, Centre for Health and Society, Medizinische Fakultät und Universitätsklinikum, Heinrich-Heine-Universität Düsseldorf, Düsseldorf, Deutschland; 3 Gesundheitsamt der Landeshauptstadt Düsseldorf, Düsseldorf, Nordrhein-Westfalen Deutschland

**Keywords:** Öffentliches Gesundheitswesen, Public Health, Aus‑, Fort- und Weiterbildung, Evidenzbasierte Praxis, Wissenstransfer, Public health administration, Public health, Education, Evidence-based practice, Translational Science, Biomedical

## Abstract

**Einleitung:**

Für eine wissenschaftsbasierte Arbeitsweise im Öffentlichen Gesundheitsdienst (ÖGD) sind tragfähige Strukturen zur Generierung praxisrelevanter Forschung sowie zur Integration selbiger in die Praxis erforderlich. Um diese Schnittstelle zu stärken, wurde im Forschungsverbund Öffentliche Gesundheit das berufsbegleitende Trainee-Rotationsprogramm „EvidenzÖGD“ entwickelt, pilotiert und evaluiert.

**Methodik:**

Die formative Prozessevaluation der 6‑monatigen Pilotierung beruhte auf einem theoriebasierten Wirkmodell und kombinierte qualitative und quantitative Methoden: Dokumentenanalysen, standardisierte Online-Befragungen und Workshops. Die Ergebnisse wurden mittels Methoden- und Datentriangulation zusammengeführt.

**Ergebnisse:**

Trainees und Mentor:innen berichteten über substanzielle Kompetenzzuwächse und identifizierten Impulse für die Verankerung eines nachhaltigen Wissenstransfers. Auf institutioneller Ebene wurden neue Kooperationsansätze und strukturelle Entwicklungen angestoßen. Gleichzeitig zeigten sich Optimierungspotenziale, etwa eine zu knappe Rotationsdauer, hoher Dokumentationsaufwand, unklare Rollenerwartungen sowie organisatorische Herausforderungen. Diese wurden systematisch aufgearbeitet und führten zur Anpassung zentraler Modellkomponenten, wie der Rotationsstruktur, zur Einführung eines einheitlichen Logbuchs, zur Ausweitung der Fortbildungsreihe und zur besseren Integration des Trainee-Forschungsprojekts.

**Diskussion:**

Das Programm wird als vielversprechender Ansatz zur Förderung wissenschaftsbasierter Praxis und zur Stärkung institutioneller Kooperationen im ÖGD bewertet. Die modulare Struktur erlaubt eine flexible Anwendung. Künftige Forschung sollte die langfristige Wirkung auf Kompetenzaufbau und die Etablierung evidenzbasierter Ansätze untersuchen.

**Zusatzmaterial online:**

Zusätzliche Informationen sind in der Online-Version dieses Artikels (10.1007/s00103-025-04160-z) enthalten.

## Abstract (en)

**Introduction:**

A science-based approach in local public health services (PHS) requires robust structures both for generating practice-relevant research and for integrating this knowledge into routine public health practice. To strengthen this interface, an academia–practice research consortium developed, piloted, and evaluated the part-time trainee-rotation program “EvidenzOGD.”

**Methods:**

The formative process evaluation of the six-month pilot was guided by a theory-based logic model and employed a mixed-methods design, including document analysis, standardized online surveys, and workshops. Findings were synthesized using method and data triangulation.

**Results:**

Trainees and mentors reported substantial gains in competencies and identified impulses for anchoring sustainable evidence transfer. At the institutional level, new forms of cooperation and structural developments were initiated. At the same time, several areas for improvement emerged, including an overly short rotation period, high documentation workload, unclear role expectations, and coordination challenges. These issues were systematically addressed, leading to adaptations of key program components, such as the rotation structure, the implementation of a unified logbook, the expansion of the training modules, and the improved integration of the trainee research project.

**Discussion:**

The program is considered a promising approach to promoting evidence-informed practice and strengthening institutional cooperation within the PHS. Its modular structure allows for flexible implementation in different local contexts. Future research should explore its long-term effects on competency development and the institutionalization of evidence-based approaches.

## Einleitung

Angesichts sich wandelnder Anforderungen und zunehmender Komplexität öffentlicher Gesundheitsaufgaben bei gleichzeitig begrenzten Ressourcen ist der Öffentliche Gesundheitsdienst (ÖGD) gefordert, gesundheitspolitische Maßnahmen und Prioritäten evidenzinformiert zu treffen und nachvollziehbar zu begründen. Aufgrund ihrer fachlichen Expertise, ihres Zugangs zu Routinedaten und ihrer Schnittstellenfunktion in der kommunalen Steuerung sind Gesundheitsämter besonders geeignet, eine zentrale Rolle in der wissenschaftsbasierten Politikberatung einzunehmen [1] – beispielsweise in der Priorisierung von Hitzeschutzmaßnahmen, der Entwicklung kommunaler Gesundheitsstrategien oder auch der Planung evidenzbasierter Infektionsschutzmaßnahmen.

Damit gesundheitsbezogene Entscheidungen wirksam und an den Bedarfen der Bevölkerung orientiert getroffen werden können, sind tragfähige Strukturen zur Generierung und Anwendung wissenschaftlicher Erkenntnisse in der Praxis erforderlich. So hebt auch das 2018 verabschiedete Leitbild für den modernen ÖGD die Bedeutung einer *wissenschaftlich fundierten Arbeitsweise *explizit hervor [2]. Unklar bleibt jedoch, wie diese im behördlichen Alltag definiert und konkret realisiert werden kann. Angelehnt an etablierte Konzepte der evidenzbasierten Medizin (EbM) und der evidenzbasierten Public Health (EbPH) werden *wissenschaftsbasierte Ansätze* oft als die systematische Zusammenführung wissenschaftlicher Erkenntnisse, fachlicher Expertise und gesellschaftlicher Wertvorstellungen verstanden [3, 4]. Aufgrund ihrer Nähe zu diversen Lebenswelten bei gleichzeitiger Verankerung in lokalen Verwaltungsstrukturen sind die Gesundheitsämter prädestiniert für die Umsetzung wissenschaftsbasierter Ansätze in der Praxis [5]. Zudem ermöglichen etablierte Schnittstellen zwischen Wissenschaft und Praxis das Erarbeiten passgenauer Lösungsansätze für kommunale Problemlagen.

In der lokalen Praxis ist die Umsetzung jedoch häufig fragmentiert. Barrieren bestehen u. a. in begrenzten personellen Kapazitäten, eingeschränkten Zugängen zu Forschungsergebnissen, fehlender politischer Rückendeckung und unzureichenden Qualifizierungs- und Austauschformaten für den Wissenstransfer zwischen Wissenschaft und Praxis [6, 7]. Hinzu kommt, dass ÖGD-relevante Fragestellungen in der Wissenschaft oft unterrepräsentiert sind und in vielen Forschungsvorhaben konkrete Implementierungsstrategien fehlen [8]. Dabei wurde der Bedarf einer systematischen Verzahnung von Wissenschaft und Praxis – etwa durch gemeinsame Projekte, integrierte Ausbildungsformate oder intermediäre Rollen – vielfach benannt [9, 10] und international erprobt [11–13]. In Deutschland erschweren bürokratische Hürden und unterschiedliche institutionelle Systemlogiken eine gleichberechtigte Zusammenarbeit [14]. So bestehen etwa Unterschiede in den Anreizstrukturen: Während in der Wissenschaft Publikationen und Drittmittel zentrale Leistungsindikatoren sind, liegt der Fokus im ÖGD auf anwendungsorientierter Politikberatung und praktischer Umsetzung [15, 16]. Ein gezielter Transfer von Forschungsergebnissen wird aktuell von der deutschen Hochschullandschaft selten gefördert oder gefordert. Hinzu kommt, dass der Austausch zwischen der eher theoriegeleiteten Public-Health-Forschung angelsächsischer Prägung und dem operativ geprägten ÖGD historisch bedingt in Deutschland lange Zeit unzureichend war [17, 18] und erst in den letzten Jahren eine verstärkte Annäherung zu beobachten ist [19].

Zur Überwindung der Wissenschafts-Praxis-Diskrepanz braucht es langfristige Kooperationen zwischen Hochschulen und Gesundheitsämtern. Der hierfür notwendige Wissenstransfer erfordert fundierte System- und Methodenkenntnisse, die vor allem durch praxisnahe Aus‑, Fort- und Weiterbildung aufgebaut werden können [20]. Mit der Facharztweiterbildung Öffentliches Gesundheitswesen (ÖGW; [21]) und dem Postgraduiertenprogramm für angewandte Epidemiologie (PAE; [22, 23]) bestehen zwar etablierte Formate, diese richten sich jedoch entweder an spezifische Berufsgruppen oder fokussieren auf spezielle Aufgabenbereiche. Es fehlt an interdisziplinären, sektorübergreifenden Programmen, die niedrigschwellig angelegt sind und den Kompetenzaufbau in der Breite ermöglichen.

Vor diesem Hintergrund wurde der Forschungsverbund Öffentliche Gesundheit, bestehend aus der Akademie für Öffentliches Gesundheitswesen (AÖGW), dem Gesundheitsamt Düsseldorf (GA-D) und der Heinrich-Heine-Universität Düsseldorf (HHU) gegründet, um praxisrelevante Forschung zu fördern, nachhaltige Wissenstransferstrukturen aufzubauen und langfristig evidenzbasierte Arbeitsweisen im ÖGD zu stärken. Als ein zentrales Projekt des Verbundes wurde das berufsbegleitende Trainee-Rotationsprogramm *EvidenzÖGD* entwickelt [24]. Ziel des Programms ist es, wissenschaftsbasierte Praxis durch Rotationsphasen zwischen Hochschulen und Gesundheitsämtern zu stärken und nachhaltige Kooperationsstrukturen an der Schnittstelle zu etablieren (Infobox 1). Das Programm wurde im Rahmen einer 6‑monatigen Pilotierung implementiert, wissenschaftlich begleitet und auf Grundlage der Evaluationsergebnisse weiterentwickelt.

Der vorliegende Beitrag berichtet über die Prozessevaluation der Pilotierung. Ziel war es, (i) die Programmumsetzung systematisch zu analysieren, (ii) die Wirkmechanismen auf Basis eines theoretischen Wirkmodells zu bewerten und (iii) Potenziale für die Übertragbarkeit zu identifizieren.

## Methodik

Im Folgenden wird eine kurze Übersicht über die Pilotierung gegeben, gefolgt von einer Darstellung der Evaluationsmethodik.

### Pilotierung des Trainee-Rotationsprogramms.

**Abb. 1:**
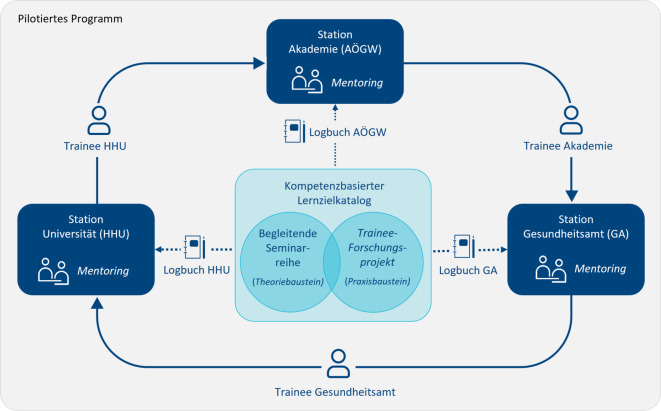
Pilotierung des Trainee-Rotationsprogramms EvidenzÖGD (04–10/2023). Abkürzungen: AÖGW = Akademie für Öffentliches Gesundheitswesen, GA-D = Gesundheitsamt Düsseldorf, HHU = Heinrich-Heine-Universität Düsseldorf

Die Pilotierung fand von April bis Oktober 2023 mit den 3 Verbundpartner:innen in Düsseldorf statt. Aus jeder Einrichtung nahm sowohl ein Trainee als auch ein:e Mentor:in teil. Die Trainees rotierten in 3 aufeinanderfolgenden 2‑monatigen Phasen zwischen den Institutionen und waren mit 50 % Vollzeitäquivalent (VZÄ) in das Programm eingebunden. Die verbleibende Arbeitszeit blieb für ihre reguläre Tätigkeit in der Basisinstitution. Formale Rahmenbedingungen wurden vorab über eine Kooperationsvereinbarung geregelt. Abb. 1 zeigt die Rotationsstruktur des pilotierten Programms.

### Prozessevaluation der Pilotierung.

**Abb. 2:**
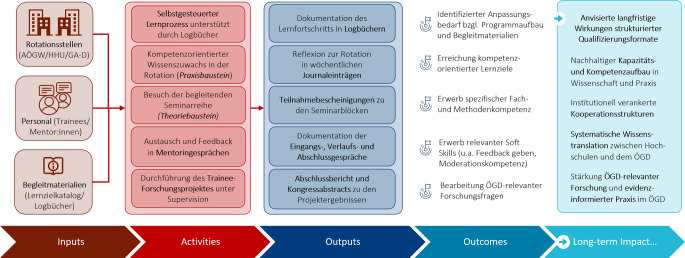
Logisches Modell zur Programmevaluation. Abkürzungen: *AÖGW* Akademie für Öffentliches Gesundheitswesen, *GA‑D* Gesundheitsamt Düsseldorf, *HHU* Heinrich-Heine-Universität Düsseldorf

Die wissenschaftliche Begleitung der Pilotphase erfolgte durch 2 Mitglieder des Forschungsverbundes, die nicht in die Konzeption oder Umsetzung des Programms eingebunden waren. Die Bewertung orientierte sich an einem logischen Modell auf Basis des „Program Evaluation Frameworks“ des Center for Disease Control (CDC; [25]), das in gemeinsamen Sitzungen der Projektsteuerungsgruppe (PSG) und des Evaluationsteams konzipiert und iterativ weiterentwickelt wurde (Abb. 2).

Das formative, prozessorientierte Evaluationsdesign [26] verfolgte das Ziel, auf Grundlage der Erfahrungen der Teilnehmenden sowie der im Wirkmodell verankerten Logik Optimierungspotenziale zu identifizieren. Im Mittelpunkt standen die 4 Programmkomponenten (I) *Rotation mit Mentoring*; (II) *Begleitmaterialien*; (III) *Fortbildungsreihe* und (IV) *Trainee-Forschungsprojekt. *Zur Datenerhebung kamen qualitative und quantitative Methoden zum Einsatz: Dokumentenanalysen (Logbücher, Journaleinträge und Protokolle), Trainee-Mentor:innen-(TM-)Workshops sowie standardisierte Online-Befragungen der Fortbildungsteilnehmer:innen. Einige Methoden wurden komponentenübergreifend eingesetzt und die Ergebnisse anschließend zusammengeführt (Methoden- und Datentriangulation; [27]). Tab. 1 gibt einen Überblick über Zielsetzung und Methodik sowie über Teilnehmende und Adressat:innen.

**Tab. 1:** Überblick über die methodischen Ansätze zur Analyse der 4 Evaluationskomponenten

Evaluations-komponente	Methode	Datenerhebung und Auswertung	Teilnehmende/Adressat:innen
i. Rotationsblöcke	Qualitativ-quantitative Dokumentenanalyse	Auswertung von Gesprächsprotokollen der leitfadengestützten Einführungs‑, Verlaufs- und Abschlussgespräche zwischen Trainees und Mentor:innen, von wöchentlichen Journaleinträgen der Trainees sowie ergänzenden Einträgen in den institutionsspezifischen Logbüchern^a^. Die Analyse erfolgte mittels qualitativer Inhaltsanalyse bezogen auf Ressourcenverfügbarkeit, Zielerreichung, Zufriedenheit und Verbesserungspotenziale durch das Evaluationsteam	Die Gesprächsprotokolle wurden von den Trainees angefertigt und mit den Mentor:innen abgestimmt
	Qualitativer TM-Workshop	In einem 3‑stündigen Präsenzworkshop diskutierten Trainees und Mentor:innen in getrennten Gruppen über Qualifikationen, Machbarkeit, Kompetenzentwicklung und Zusammenarbeit während der Rotation. Zur Vorbereitung erhielten die Teilnehmenden einen semistrukturierten Protokollleitfaden (siehe Onlinematerial 1). Beide Workshop-Gruppen wurden jeweils von einem Evaluationsteammitglied moderiert, mittels Fotodokumentation protokolliert und anschließend qualitativ-inhaltsanalytisch ausgewertet	An dem Workshop nahmen die Trainees und Mentor:innen teil. Nach einer gemeinsamen Einführung diskutierten beide Teilnehmergruppen separat
ii. Begleitmaterialien	Qualitativ-quantitative Dokumentenanalyse	In den institutionsspezifischen Logbüchern dokumentierten die Trainees zusätzlich zu ihrem Lernfortschritt auch Herausforderungen und Verbesserungsvorschläge. Diese Einträge wurden vom Evaluationsteam qualitativ-inhaltsanalytisch ausgewertet und es wurden konkrete Verbesserungsvorschläge an die Projektsteuerungsgruppe (PSG) weitergegeben	Die institutionsspezifischen Logbücher wurden federführend von den Trainees ausgefüllt; Mentor:innen ergänzten die Lernzielerreichung
	Qualitativer TM-Workshop	Feedback im TM-Workshop zu Logbüchern und Handbuch wurde vom Evaluationsteam aufgegriffen und separat analysiert. Im Mittelpunkt standen Verständlichkeit, Handhabbarkeit und Relevanz der Materialien zur Lernzielerreichung	Das Evaluationsteam bestand aus 2 Mitgliedern des Forschungsverbundes
iii. Seminarreihe	Quantitative Online-Befragung	Nach jedem Veranstaltungsblock wurden standardisierte Online-Befragungen mittels LimeSurvey durchgeführt. Bewertet wurden u. a. Umfang, Struktur, Verständlichkeit, Relevanz und Neuheitsgehalt der Veranstaltungen (siehe Onlinematerial 2). Teilnehmende erhielten per E‑Mail eine Einladung zur Evaluation. Offene Fragen wurden qualitativ-inhaltsanalytisch ausgewertet, während kategoriale Daten in Häufigkeitstabellen deskriptiv vom Evaluationsteam zusammengefasst wurden	Die Online-Befragung wurde allen Teilnehmenden der begleitenden Seminarreihe zugesandt. Dies umfasste auch die Trainees und Mentor:innen
iv. Trainee-Forschungsprojekt	Qualitativ-quantitative Dokumentenanalyse	Protokolle des Forschungskolloquiums, Sitzungen der PSG, wöchentliche Journaleinträge der Trainees sowie Logbuchauszüge wurden vom Evaluationsteam mittels qualitativer Inhaltsanalysen ausgewertet. Betrachtet wurden insbesondere Machbarkeit, Ressourcenbedarf und Optimierungsmöglichkeiten	Am Forschungskolloquium nahmen Trainees und Mentor:innen teil. Die PSG setzte sich aus Mitgliedern des Forschungsverbundes Öffentliche Gesundheit zusammen
	Qualitativer TM-Workshop	Vertiefte Rückmeldungen zur Umsetzung und organisatorischen Einbindung des Trainee-Forschungsprojekts im TM-Workshop wurden qualitativ-inhaltsanalytisch vom Evaluationsteam ausgewertet	Das Evaluationsteam bestand aus 2 Mitgliedern des Forschungsverbundes

^*a*^ *Institutionsspezifische Logbücher* bezeichnen einrichtungsspezifisch erstellte Dokumentationsformate mit festgelegten Inhalten und Lernzielen, die durch die Trainees pro Rotationsblock ausgefüllt werden. Im Rahmen der Pilotierung wurden insgesamt 9 Logbücher ausgefüllt und analysiert (3 pro Trainee)

*TM* Trainees und Mentor:innen, *PSG* Projektsteuerungsgruppe

Quantitative Daten wurden deskriptiv mittels Häufigkeitsauszählungen in Microsoft Excel analysiert, qualitative Daten inhaltsanalytisch nach Mayring [28] in MAXQDA 24. Auf Basis des erhobenen Textmaterials wurden induktiv Kategorien entwickelt, zu einem Kodierleitfaden zusammengeführt (s. Onlinematerial 3) und durch das Evaluationsteam auf das gesamte Material angewendet. Die Ergebnisse wurden anschließend in 3 halbtägigen vom Evaluationsteam durchgeführten und dokumentierten Evaluationsworkshops mit Mitgliedern der PSG sowie den beteiligten Trainees und Mentor:innen diskutiert und in einem abschließenden Fachtag mit allen validiert.

### Ethik und Qualitätssicherung.

Die Studie wurde in Übereinstimmung mit der Deklaration von Helsinki [29] durchgeführt und durch die Ethikkommission der Medizinischen Fakultät der HHU genehmigt. Zur Sicherstellung von Intersubjektivität wurde ein reflexiver, interdisziplinärer Ansatz gewählt. Monatliche Treffen der PSG sowie regelmäßige Konsultationen mit externen Expert:innen ermöglichten eine kritische Auseinandersetzung mit methodischen Entscheidungen und Ergebnisinterpretationen. Angaben zum Forschungsteam finden sich in Onlinematerial 4.

Die Beschreibung von Methodik und Ergebnissen erfolgte in Anlehnung an die TIDieR-Leitlinie [30] und die COREQ-Checkliste ([31]; Onlinematerial 5 und 6).

## Ergebnisse

An der Pilotphase nahmen 3 TM-Tandems teil, jeweils eines pro Institution. Die Trainees verfügten über erste Berufserfahrung oder befanden sich in der Abschlussphase ihres Masterstudiums. Alle Mentor:innen verfügten über einschlägige Praxis- bzw. Forschungserfahrung sowie einen akademischen Hintergrund. In beiden Gruppen waren jeweils 2 Frauen und ein Mann vertreten.

Die Auswertung der Logbücher zur Analyse der Programmumsetzung (Ziel 1) zeigte, dass ein Großteil der vorgesehenen Lernziele während der Rotation erreicht wurde. Abweichungen ergaben sich v. a. in Bezug auf das Trainee-Forschungsprojekt, das aufgrund von Verzögerungen im Projektstart nicht in vollem Umfang abgeschlossen werden konnte. Die Analyse der TM-Gespräche, Protokolle der Forschungskolloquien und PSG-Sitzungen bestätigte eine weitgehend planmäßige Umsetzung des Programms.

Die Bewertung der Wirkmechanismen anhand der beobachtbaren Outcomes (Ziel 2) zeigte positive Effekte auf individueller wie institutioneller Ebene. Im TM-Workshop berichteten Trainees und Mentor:innen von einem substanziellen Lernzuwachs. Die Trainees beschrieben eine Weiterentwicklung ihrer fachlichen, methodischen und kommunikativen Kompetenzen sowie ein vertieftes Verständnis für die unterschiedlichen Systemlogiken von Wissenschaft und ÖGD. Die Verbindung von theoretischer Wissensvermittlung und praxisnaher Anwendung wurde als besonders lernförderlich beschrieben. Auf Mentor:innenseite wurden Kompetenzzuwachs in den Bereichen Gesprächs- und Mitarbeiterführung sowie eine vertiefte Reflexion des eigenen professionellen Handelns hervorgehoben. Beide Gruppen berichteten von einer gesteigerten Sensibilisierung für evidenzbasierte Arbeitsweisen und von neuen Impulsen für inter- und intrainstitutionelle Wissenstransferprozesse.

Auch auf institutioneller Ebene wurden positive Veränderungen festgestellt: Im Rahmen des abschließenden Fachtags – an dem neben den Trainees und Mentor:innen auch Führungskräfte aller 3 Institutionen teilnahmen – wurde betont, dass das Programm über die individuelle Qualifizierung hinaus strukturelle Entwicklungen angestoßen habe. Die Einbindung von Trainees in die eigene Institution wurde mehrfach als Anlass genutzt, um etablierte Abläufe zu reflektieren (z. B. bezüglich des Onboardings neuer Mitarbeitenden), neue fachliche Perspektiven zu integrieren (z. B. durch spezifische Fach- und Soft Skills der Trainees) und die Zusammenarbeit zu intensivieren (z. B. im Rahmen eines neu etablierten Forschungskolloquiums). Hochschulseitig wurde die praktische Anwendbarkeit von Forschungsergebnissen hervorgehoben. Der regelmäßige Austausch wurde zudem positiv für die Entwicklung neuer Forschungsfragen erachtet. Seitens des Gesundheitsamtes wurde die Möglichkeit zur Nachwuchsqualifizierung und -gewinnung sowie zur Etablierung systematischer Wissens- und Transferstrukturen positiv hervorgehoben. Die Akademie identifizierte Potenziale für eine systematische Weiterentwicklung kommunaler Aus‑, Fort- und Weiterbildungsstrukturen sowie Optionen für eine zukünftige Ausweitung des Trainee-Rotationsprogramms.

Insgesamt wurde die pilotierte Version des Programms von allen Beteiligten als positiv und gewinnbringend für den Aufbau nachhaltiger Strukturen bewertet. Gleichzeitig konnten im Rahmen der Begleitevaluation Optimierungspotenziale für die Weiterentwicklung des Programms identifiziert werden. Tab. 2 fasst die zentralen Herausforderungen zusammen und stellt die erarbeiteten Verbesserungsvorschläge gegenüber.

### (i) Rotationsblöcke und Mentoring.

Die Rotation in unterschiedlichen institutionellen Kontexten und das unterstützende Mentoring wurden von den Trainees als besonders gewinnbringend bewertet. Optimierungsbedarf bestand v. a. in strukturellen Aspekten der Rotation [1]. (1) Die angestrebte Rotationsdauer von 6 Monaten bei 50 % VZÄ wurde als zu knapp für eine umfassende Lernzielerreichung eingeschätzt. Empfohlen wurde eine Verlängerung auf 12 Monate. (2) Der Koordinationsaufwand der Rotation zwischen 3 Institutionen wurde gerade zu Beginn als recht hoch bewertet. Gleichzeitig war die Beteiligung der AÖGW an der Trainee-Rotation nicht dauerhaft vorgesehen, sondern diente v. a. der Gewinnung praktischer Erfahrungswerte, um künftige Rotationen angemessen begleiten zu können (z. B. in der Durchführung begleitender Schulungen). Entsprechend wurde empfohlen die Zahl der Rotationsinstitutionen von 3 auf 2 zu reduzieren. (3) Zur Erleichterung der Zusammenarbeit wurden das frühzeitige Festlegen von Rotationswochentagen und regelmäßigen Austauschformaten (z. B. Jour fixe, Forschungskolloquien) angeregt sowie klare Vertretungsregelungen vorgeschlagen. (4) Trainees und Mentor:innen berichteten von Unklarheiten bzgl. der eigenen sowie gegenseitigen Rollenerwartung zu Beginn der Rotation. Es wurden einführende TM-Schulungen zu Beginn der Programmteilnahme angeregt, um die Abläufe und Herangehensweisen (z. B. die Arbeit mit dem Logbuch) abzustimmen.

### (ii) Begleitmaterialien.

Das Logbuch wurde als hilfreiches Instrument zur Lernsteuerung eingeschätzt, insbesondere in Verbindung mit dem integrierten Lernzielkatalog und der darin verankerten Kontextzuordnung (Lernzielerreichung in der Fortbildungsreihe, während der Rotation oder im Trainee-Forschungsprojekt). Verbesserungspotenziale ergaben sich v. a. in der Reduktion des Dokumentationsaufwandes und in der Handhabbarkeit: (1) Die parallele Nutzung von 3 Logbüchern pro Trainee (eines pro Rotationsphase) wurde als aufwendig bewertet. (2) Es zeigte sich ein Bedarf, einzelne Lernziele hinsichtlich Klassifizierung und Kompetenzniveau zu überarbeiten. (3) Während der Pilotierung erfolgte die Dokumentation der Lernzielerreichung im Logbuch digital über einen Cloud-Speicher, auf den Trainees und Mentor:innen Zugriff hatten. Um die Lernzielerreichung einfacher dokumentieren und digital bestätigen zu können, wurde die Entwicklung eines E‑Logbuchs mit hierarchischem Rechtemanagement vorgeschlagen. Zudem wurde die Entwicklung eines Programmhandbuchs für interessierte Kommunen angeregt – inkl. Empfehlungen zu (4) Rotationsdauer, (5) organisatorischer Umsetzung (z. B. Musterkooperationsvereinbarungen, Onboarding-Checklisten) sowie (6) standardisierter Rollen- und Kompetenzprofile für Trainees und Mentor:innen.

### (iii) Fortbildungsreihe.

Die Teilnahme an den Online-Fortbildungsblöcken war für Trainees verpflichtend, für Mentor:innen fakultativ und stand weiteren Interessierten aus Wissenschaft und Praxis offen. An den 3 Blöcken nahmen insgesamt 336 Personen teil; darunter Vertreter:innen aus dem kommunalen ÖGD, von Landes- und Bundesbehörden sowie aus der Wissenschaft. Die Evaluation (*n* = 167) zeigte eine hohe Zufriedenheit mit Inhalt und Format. Insgesamt wurde der Reihe eine hohe praktische Relevanz zugeschrieben und die Durchführung wurde als praxisnah und methodisch fundiert bewertet. Weiterentwicklungsmöglichkeiten zeigten sich insbesondere in Bezug auf die didaktische Ausgestaltung und den Umfang: (1) Es wurde angeregt, einzelne Blöcke zu entzerren und (2) bestimmte Themen mit weiteren Praxisübungen zu vertiefen.

### (iv) Trainee-Forschungsprojekt.

Das Trainee-Forschungsprojekt wurde von allen Beteiligten als zentrales und gewinnbringendes Element hervorgehoben. Trotz Verzögerungen im Projektstart konnten die Trainees das vom Gesundheitsamt eingebrachte Thema zur *medizinischen Versorgung und deren Inanspruchnahme bei wohnungslosen Personen in Düsseldorf* erfolgreich bearbeiten. Die Ergebnisse fanden Eingang in die strategische Planung des Gesundheitsamtes, wurden lokal sowie wissenschaftlich disseminiert und nach Projektabschluss weiterverwendet. Optimierungspotenziale betrafen vor allem die zeitliche Planung und methodische Ausrichtung: (1) Unklare Zuständigkeiten zu Beginn der Rotation verzögerten die Festlegung der gemeinsamen Forschungsfragen. Um ausreichend Zeit für die Bearbeitung des Forschungsthemas zu haben, wurde empfohlen, die Themenfindung künftig bereits vor Beginn der Rotation anzuvisieren. (2) Als besonders anschlussfähig für den bilateralen Wissenstransfer wurde der methodische Zugang über Evidenzsynthesen bewertet. (3) Die Logbuchanalysen zeigten Unklarheiten bzgl. der Integration des Projektes in den Lernzielkatalog auf.

**Tab. 2:** Zentrale Herausforderungen und Anpassungsmöglichkeiten der Trainee-Rotation

Evaluationskomponente	Zentrale Herausforderungen	Identifizierte Anpassungsmöglichkeiten
i. Rotationsblöcke	Knappe zeitliche Ressourcen (Rotation und Projekt bei 50 % Vollzeitäquivalent)	Strukturelle Änderungen innerhalb der Rotationsblöcke:
		(1) Verlängerung des Rotationszeitraums von 6 auf 12 Monate
	Hoher Koordinationsaufwand der Rotation zwischen 3 Institutionen	(2) Reduzierung der Zahl der Rotationsinstitutionen von 3 auf 2 (Gesundheitsamt und Hochschule)
	Unklare Strukturen (feste Rotationszeiten, Vertretungsregelungen)	(3) Erarbeitung von Empfehlungen zur Ausgestaltung und zum Turnus von Austauschformaten zwischen Trainees und Mentor:innen (z. B. Jour fixe)
	Bedarf an vorbereitenden Schulungen für Trainees und Mentor:innen	(4) Entwicklung von Trainee-Mentor:innen-(TM-)Schulungen zur Rotationsvorbereitung
ii. Begleitmaterialien	Hoher Dokumentationsaufwand durch institutionsspezifische Logbücher	Überarbeitung des *Logbuchs *und des *kompetenzorientierten Lernzielkatalogs*:
	Unklare Zielzuordnungen und Lernzielformulierungen bei einzelnen Lernzielen	(1) Zusammenführung von 3 institutionsspezifischen Logbüchern zu einem einheitlichen, institutionsübergreifenden Logbuch
	Unrealistische Kompetenztiefe einzelner Lernziele	(2) Überarbeitung des Kompetenzniveaus einzelner Lernziele
	Fehlende digitale Unterstützung zur Lernverfolgung und Lernzielbestätigung	(3) Entwicklung eines E‑Logbuchs zur digitalen Dokumentation
	Mangel an adaptiven Empfehlungen für verschiedene kommunale Kontexte	Ausarbeitung eines umfassenden *Programmhandbuchs *als Handreichung für interessierte Kommunen
	Fehlende Informationen zu Rollenprofilen, Umsetzung und Kooperationsstrukturen	(1) Erarbeitung von Empfehlungen für die Durchführung der Trainee-Rotation EvidenzÖGD (inkl. Dauer der Rotationsphasen)
	Bedarf an Vorlagen und Checklisten zur praktischen Umsetzung des Trainee-Rotationsprogramms	(2) Erarbeitung von Empfehlungen für organisatorische Rahmenbedingungen (inkl. Musterkooperationsvereinbarungen, Onboarding-Checklisten)
	Unklare Rollenerwartungen an Trainees und Mentor:innen	(3) Ausarbeitung detaillierter Rollen- und Kompetenzprofile für Trainees und Mentor:innen
iii. Begleitseminar	Überarbeitungsbedarf der Inhalte zur besseren Abdeckung der Lernziele	Erweiterung und Anpassung des Begleitseminars:
		(1) Integration zusätzlicher Themenblöcke zur besseren Abdeckung der überarbeiteten Lernziele
	Wunsch nach mehr Praxisbezug und interaktiven Elementen	(2) Erweiterung der didaktischen Formate
iv. Trainee-Forschungsprojekt	Verzögerter Projektstart durch Abwesenheiten und unklare Verantwortlichkeiten	Integration des Trainee-Forschungsprojekts in das Rotationsprogramm:
	Hoher Zeitaufwand für Themenfindung und Formulierung der Forschungsfrage	(1) Erarbeitung von Empfehlungen zur frühzeitigen Themenfindung und Forschungsfrageentwicklung
	Bedarf an methodischer Fokussierung (z. B. Evidenzsynthesen)	(2) Vorschlag, Evidenzsynthesen als geeignete methodische Grundlage zu nutzen
	Unklare Integration des Projekts in Lernzielkatalog und Logbuch	(3) Explizite Verankerung der Forschungsprojekte als eigenständige Komponente im Lernzielkatalog und Logbuch

### Triangulation der Evaluationsergebnisse zur Adaption des Trainee-Rotationsprogramms

**Abb. 3:**
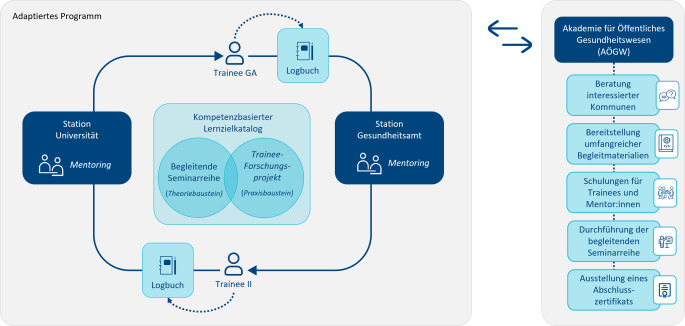
Adaptiertes Trainee-Rotationsprogramm

Die Zusammenführung der Ergebnisse aus den 4 Evaluationskomponenten ergab eine ausreichende Datensättigung. Die in Tab. 2 zusammengefassten Erkenntnisse flossen in die Weiterentwicklung des Programms ein. Im Fokus standen dabei insbesondere die Umsetzbarkeit im beruflichen Alltag sowie die Übertragbarkeit auf andere kommunale Kontexte (Ziel 3). Zentrale Anpassungen umfassten die Reduktion der Rotationsinstitutionen von 3 auf 2, die Zusammenführung der 9 institutionsspezifischen Logbücher zu einem institutionsübergreifenden Dokument sowie die Überarbeitung des Lernzielkatalogs mitsamt Reduktion der Lernziele von 89 auf 75. Die begleitende Seminarreihe wurde entsprechend angepasst und auf 7 Blöcke erweitert. Das Trainee-Forschungsprojekt wurde als eigenständige Komponente zur Erreichung spezifischer Lernziele in das Logbuch integriert. Für die Implementierung in weiteren Kommunen wurde ein Programmhandbuch erarbeitet [32], das Empfehlungen zur Durchführung sowie Vorlagen zu organisatorischen Rahmenbedingungen (inkl. Musterkooperationsvereinbarungen und Onboarding-Checklisten) und Rollen- und Kompetenzprofilen für Trainees und Mentor:innen enthält. Abb. 3 zeigt das adaptierte Trainee-Rotationsprogramm.

## Diskussion

Eine wissenschaftsbasierte Aufgabenwahrnehmung im ÖGD erfordert, dass für Praxisfragen tragfähige Lösungen erarbeitet, implementiert und evaluiert werden. Die Pilotierung des Trainee-Rotationsprogramms zeigt, dass das Programm ein vielversprechender Ansatz ist, um Wissenstransferkompetenzen zu fördern und Wissenschafts-Praxis-Kooperationen institutionell zu stärken.

Der berufsbegleitende Rotationsansatz wurde als praktikable Lösung zur Qualifizierung an der Schnittstelle zwischen Wissenschaft und Praxis betrachtet. Besonders die Verknüpfung verschiedener Lernorte mit definierten Lernzielen, die Möglichkeit zum selbstgesteuerten Kompetenzerwerb sowie die Einbindung in praxisrelevante Forschungsprojekte erschienen zentral für die Akzeptanz des Programms. Die Trainees berichteten über substanzielle fachliche, methodische und kommunikative Lerngewinne, während Mentor:innen hilfreiche Impulse in den Bereichen Coaching und Gesprächsführung wahrnahmen. Institutionell konnten neue Kooperationsansätze, Nachwuchsförderung und institutionsinterne sowie -übergreifende Austauschprozesse angestoßen werden. Die Programmstruktur wurde für zukünftige Rotationen angepasst – insbesondere mit Blick auf zeitliche Rahmenbedingungen, Rotationsorganisation und die Rollen der beteiligten Institutionen –, um eine bessere Verzahnung der Module und Umsetzbarkeit im Berufsalltag zu gewährleisten.

### Das weiterentwickelte Trainee-Rotationsprogramm im Kontext bestehender Programme

Das Trainee-Rotationsprogramm zählt zu den ersten Programmen in Deutschland, die gezielte Kompetenzentwicklung systematisch mit dem Aufbau lokaler Wissenstransferprozesse verbinden. Es adressiert zentrale Herausforderungen, wie die begrenzte Verzahnung von Wissenschaft und Praxis im ÖGD, fehlende Anreizstrukturen für anwendungsorientierte Forschung und wissenschaftsbasierte Praxis im ÖGD sowie knappe Ressourcen auf beiden Seiten [7]. Im Vergleich zu etablierten Qualifizierungsformaten (z. B. Facharztweiterbildung, PAE, Master Public Health – MPH) liegt der Fokus des Trainee-Rotationsprogramms auf der kommunalen Schnittstelle zwischen Praxis und Wissenschaft. Konzeptionell reiht es sich in internationale Entwicklungen wie das 2024 von der EU initiierte *Health Union Fellowship* ein [33]. Beide Programme verfolgen das Ziel, durch arbeitsplatzintegriertes Lernen, Mentoring und gemeinsame Forschungsprojekte anwendungsorientierte Kompetenzen an der Schnittstelle von Wissenschaft und Praxis zu fördern.

Das pilotierte Programm zeigt exemplarisch, wie berufsbegleitende, rotierende Lernformate zur Überwindung institutioneller „Silos“ beitragen können. Es ergänzt damit die bestehende Ausbildungslandschaft im ÖGW und setzt internationale Empfehlungen zur Stärkung wissenschaftsbasierter Praxis in komplexen Systemen um. Vor dem Hintergrund des steigenden Bedarfs an kontextsensiblen, wissenschaftsbasierten Entscheidungsstrukturen im ÖGD bieten berufsbegleitende Programme eine vielversprechende Option: Sie lassen sich in bestehende Arbeitsrealitäten integrieren und fördern den Aufbau wissenschaftsbasierter Handlungskompetenz auf beiden Seiten.

### Chancen und Herausforderungen

Das Trainee-Rotationsprogramm adressiert eine zentrale Lücke im deutschen Qualifizierungssystem für den ÖGD: den Mangel an strukturierten, praxisnahen Weiterbildungsangeboten im Bereich Wissenstransfer – ein Aspekt, dessen Fehlen von Nachwuchskräften wiederholt kritisiert wurde [34, 35]. Durch die Einbindung der Trainees profitieren Gesundheitsämter unmittelbar von methodischem Know-how und frischen Impulsen wodurch etablierte Arbeitsprozesse reflektiert werden können. Gleichzeitig können organisationsbezogene Lernprozesse angestoßen und strategische Weiterentwicklungen, etwa eine engere Anbindung an Hochschulen, gefördert werden. Aus Hochschulsicht bieten etablierte Partnerschaften Zugang zu kommunalen Daten und Einblick in Bedarfe und Steuerungslogiken, wodurch kontextbezogene Forschungsvorhaben gezielt entwickelt werden können. Damit kann das Programm die strategische Ausrichtung auf Transferaufgaben im Rahmen der „Third Mission“ unterstützen und zugleich die Sichtbarkeit wissenschaftlicher Beiträge in der öffentlichen Gesundheit stärken. Darüber kann es neue Rollenprofile für wissenschaftlich qualifizierte Fachkräfte schaffen – vergleichbar mit der im internationalen Raum etablierten Funktion von Knowledge-Brokern [36] – und zur langfristigen Verankerung wissenschaftsbasierter Praxis im ÖGD beitragen.

Über die in der Evaluation identifizierten Optimierungspotenziale hinaus traten im Zuge der laufenden Skalierung 3 zentrale Herausforderungen für die Umsetzung des Modells in unterschiedlichen kommunalen Kontexten hervor, die nachfolgend kurz diskutiert werden. Diese Einschätzungen basieren auf Diskussionen bei Fachkongressen, Rückmeldungen aus der Praxis sowie ersten Umsetzungserfahrungen.

#### (1) Ressourcen.

Die vorgesehene Freistellung im Umfang von 50 % VZÄ erfordert eine Umstrukturierung der Aufgabenverteilung und kann für kleinere Gesundheitsämter und Hochschulen eine Hürde darstellen – insbesondere vor dem Hintergrund des 2026 auslaufenden ÖGD-Pakts [37]. Neben der Entsendung der Trainees sind auch Kapazitäten für Mentoring, Koordination und notwendige Infrastruktur einzuplanen. Gleichwohl ist das Programm im Vergleich zu etablierten Weiterbildungen wie dem MPH (3–4 Semester), dem PAE (24 Monate) oder der Facharztweiterbildung ÖGW (720 Theoriestunden) ressourcenschonender konzipiert. Der berufsbegleitende Charakter ermöglicht es zudem, den Dienstbetrieb mit reduziertem Stellenanteil aufrechtzuerhalten und gleichzeitig gezielt Kompetenzen aufzubauen.

#### (2) Lokale Umsetzbarkeit.

Nicht alle Kommunen verfügen über etablierte Hochschulkooperationen oder eigene Forschungskapazitäten. Unterschiedliche institutionelle Voraussetzungen erfordern daher flexible Implementierungsstrategien. Hier liegt eine zentrale Stärke des Programms: Die modulare Struktur erlaubt passgenaue Umsetzungen – etwa in Form bilateraler Rotationen zwischen Gesundheitsämtern und Hochschulen oder durch die Einbindung von Landesinstitutionen. Die EvidenzÖGD-Toolbox sowie das begleitende Handbuch bieten konkrete Umsetzungshilfen bei der individuellen Adaption [32, 38].

#### (3) Politische Rahmensetzung.

Für eine nachhaltige Etablierung vor Ort bedarf es klarer Zuständigkeiten, strategischer Priorisierung und langfristiger Finanzierung. Internationale Studien zeigen, dass fehlendes institutionelles Commitment zu den größten Barrieren beim Aufbau funktionierender Wissenstransferstrukturen gehört [9]. Die Erfahrungen aus der Pilotierung bieten hier konkrete Argumentationsgrundlagen: Berufsbegleitende Rotationsprogramme können zur Fachkräftebindung beitragen, evidenzbasierte Steuerungskompetenzen stärken und praxisrelevante Fragestellungen systematisch aufgreifen. Kommunen, die solche Programme implementieren, entwickeln damit nicht nur essenzielle Partnerschaften, sondern positionieren sich auch strategisch als zukunftsfähig im ÖGW.

### Stärken und Limitationen der Evaluation

Die Evaluation der Pilotierung stützt sich auf ein theoriebasiertes Wirkmodell und eine Methodentriangulation aus Dokumentenanalyse, Workshops und standardisierten Befragungen. Durch die Einbindung aller relevanten Akteursgruppen – Trainees, Mentor:innen und institutionelle Vertreter:innen – konnten individuelle wie organisationale Perspektiven berücksichtigt werden.

Zugleich unterliegt die Evaluation folgenden Einschränkungen: (1) Es handelt sich um die Prozessevaluation eines erstmalig erprobten Programms an einem einzelnen Standort mit entsprechend begrenzter Fallzahl (*n* = 3 Tandems). Die Ergebnisse sind daher nicht ohne Weiteres zu verallgemeinern. Dennoch weisen sie auf eine grundsätzlich gute Umsetzbarkeit und hohe Akzeptanz des Ansatzes hin. Der Einsatz verschiedener Erhebungsformate – etwa anonymer Rückmeldungen im Onlinesurvey oder schriftlicher Logbuchkommentare sowie offener Formate (z. B. Workshops) – stärkt die Aussagekraft der Ergebnisse. (2) Eine externe Evaluation war aus Ressourcengründen nicht realisierbar. Um potenzielle Verzerrungen zu minimieren, wurde die Evaluation von 2 nicht am Programmdesign oder der Umsetzung beteiligten Mitgliedern des Forschungsverbunds durchgeführt. Zur Förderung der externen Validität wurden Zwischenergebnisse zudem auf wissenschaftlichen Kongressen präsentiert [39–42]. (3) Die individuelle Umsetzung an einem Standort ist nicht zwingend übertragbar auf einen anderen Standort und wird Anpassungen erfordern. Die EvidenzÖGD-Toolbox bietet hierfür ein praxistaugliches Instrument [38].

## Fazit

Der Artikel berichtet über die Evaluation und Weiterentwicklung des berufsbegleitenden Trainee-Rotationsprogramms *EvidenzÖGD*. Ziel ist es, Fachkräften theoretische Grundlagen und praktische Kompetenzen für den Aufbau nachhaltiger Wissenstransferprozesse an der Schnittstelle zwischen Wissenschaft und Praxis im ÖGD zu vermitteln. Die Ergebnisse der Pilotphase zeigen, dass berufsbegleitende Qualifikationskonzepte gut geeignet sind, um wissenschaftsbasierte Handlungskompetenzen aufzubauen und lokale Kooperationsstrukturen zu stärken.

Das weiterentwickelte Programm bietet durch seine modulare Struktur eine praxistaugliche Grundlage für die kontextspezifische Anwendung in weiteren Kommunen. Mit Eintritt in die Weiterentwicklungsphase besteht Forschungsbedarf zur langfristigen Wirkung und zukünftige Studien sollten prüfen, inwieweit durch das Programm objektivierbare Kompetenzzuwächse erzielt und evidenzbasierte Arbeitsweisen langfristig etabliert werden.

### Infobox Das Trainee-Rotationsprogramm EvidenzÖGD

Das berufsbegleitende Trainee-Rotationsprogramm *EvidenzÖGD* qualifiziert Fachkräfte an der Schnittstelle zwischen Wissenschaft und Praxis für zentrale Aufgabenfelder im bilateralen Wissenstransfer – darunter Netzwerk- und Gremienarbeit, Wissensmanagement, Projekt- und Changemanagement, Capacity Building sowie im Aufbau nachhaltiger Transferstrukturen [24]. Die Qualifizierung erfolgt in 2 eng verzahnten Bausteinen: (1) einem *Theoriebaustein*, bestehend aus einer Fortbildungsreihe mit mehreren Modulen zur Vermittlung fachlicher und methodischer Grundlagen, sowie (2) einem *Praxisbaustein*, bestehend aus einer Praxisphase mit institutionenübergreifender Rotation und einem anwendungsbezogenen Trainee-Forschungsprojekt. Ein strukturierter Lernzielkatalog mit 75 Lernzielen, differenziert nach den 3 Niveaustufen des *Nationalen Kompetenzbasierten Lernzielkatalogs Medizin *(NKLM) – (1) Faktenwissen, (2) Handlungs- und Begründungswissen sowie (3) Handlungskompetenz – [44], bildet die Grundlage für den Kompetenzaufbau.

Der Lernprozess ist überwiegend selbstgesteuert und wird durch ein Logbuch unterstützt, das als zentrales Instrument zur Planung, Reflexion und Dokumentation dient [45]. Es verknüpft jedes Lernziel mit den entsprechenden Programmbausteinen – Seminarreihe, Praxisrotation und Forschungsprojekt – und dient so als zentrales Nachschlage- und Arbeitsdokument. Erfahrene Mentor:innen begleiten den Prozess und fördern den professionsübergreifenden Austausch.

Konzipiert wurde das Programm auf Basis einer vom Forschungsverbund Öffentliche Gesundheit entwickelten Toolbox [38], die auf einer Analyse bestehender Kooperationsstrukturen [46] sowie zentraler förderlicher und hinderlicher Faktoren für nachhaltige Forschungskooperationen beruht [14]. Die Toolbox stellt ein praxisorientiertes Instrument zur Entwicklung kontextsensibler Fortbildungsformate dar, welches auch für die Adaption des EvidenzÖGD-Programms in andere Kontexte genutzt werden kann. Sämtliche Materialen – einschließlich Toolbox, Lernzielkatalog und ein begleitendes Handbuch – sind als Open-Access-Publikationen verfügbar [24].

## Supplementary Information

Zusätzliches Onlinematerial- Onlinematerial 1: Leitfaden TM-Workshop- Onlinematerial 2: Leitfaden Online-Befragung- Onlinematerial 3: Coding Frame- Onlinematerial 4: Zusammensetzung des Forschungsteams- Onlinematerial 5: TIDIER Checklist- Onlinematerial 6: COREQ Checklist

## Data Availability

Die während der vorliegenden Studie erzeugten und analysierten Datensätze sind zur Wahrung des Datenschutzes nicht öffentlich zugänglich. Auf begründete Anfrage können sie in reduzierter, anonymisierter Form bei der korrespondierenden Autorin angefragt werden.
