# Supplementary material for: Bridging research and practice in public health services: lessons learned from the EvidenzOGD trainee rotation program
Source: Bundesgesundheitsblatt Gesundheitsforschung Gesundheitsschutz. 2025 Dec 1;69(1):23–33. [Article in German] doi: 10.1007/s00103-025-04160-z (PMC12764494; doi:10.1007/s00103-025-04160-z)
Supplement: Supplementary file 1 — Zusätzliches Onlinematerial- Onlinematerial 1: Leitfaden TM-Workshop- Onlinematerial 2: Leitfaden Online-Befragung- Onlinematerial 3: Coding Frame- Onlinematerial 4: Zusammensetzung des Forschungsteams- Onlinematerial 5: TIDIER Checklist- Onlinematerial 6: COREQ Checklist [file 103_2025_4160_MOESM1_ESM.pdf]

Zusätzliches Onlinematerial

- Onlinematerial 1: Leitfaden TM-Workshop
- Onlinematerial 2: Leitfaden Online-Befragung
- Onlinematerial 3: Coding Frame
- Onlinematerial 4: Zusammensetzung des Forschungsteams
- Onlinematerial 5: TIDIER Checklist
- Onlinematerial 6: COREQ Checklist

Angaben zu den Referenzen befinden sich im Originalartikel.

Onlinematerial 1. Vorbereitende Leitfragen zum Trainee Mentor:innen (TM)-Workshop

Themenschwerpunkte und Fragen zur Vorbereitung

1. Qualifikation und Kompetenzen

   Erläuterung: Qualifikation meint die fachliche Ausbildung. Kompetenzen beziehen sich auf Fähigkeiten losgelöst von der Qualifikation, z.B. Erfahrungen, Softskills oder Wissen.

   Fragen:
   - Was sind deine Qualifikationen?
   - Welche Qualifikation(en) siehst du als Mindestanforderung für Mentor:innen und welche für Trainees?
   - Welche Kompetenzen sollten Mentor:innen und welche Trainees mitbringen?
   - Falls dir in deiner Rolle als Trainee oder Mentor:in etwas gefehlt hat bzgl. Qualifikation und / oder Kompetenzen, was war das?

2. Machbarkeit und Ressourcen

   Erläuterung: Machbarkeit und Ressourcen (z.B. finanziell, zeitlich, technisch, personell) fokussieren die Integrationsfähigkeit in den Arbeitsalltag. Es geht auch um einen Ist-Soll-Abgleich.

   Fragen:
   - Was hast du für deine Rolle als Trainee oder Mentor:in an Ressourcen gebraucht?
   - Standen dir die Ressourcen zur Verfügung, die du gebraucht hast? (Soll vs. Ist)
   - Wie war die Umsetzung generell vereinbar mit deinem regulären Arbeitsalltag?
   - Wie viel Zeit hast du investiert? (z.B. Wochendurchschnitt in Stunden)
   - Welche Tipps hast du für zukünftige Mentor:innen, Trainees, Institutionen?
   - Wie zufrieden bist du generell mit der Machbarkeit & den Ressourcen?

3. Kompetenzsteigerung

   Erläuterung: Kompetenzsteigerung bezieht sich sowohl auf Kompetenzerwerb, als auch auf die Weiterentwicklung bestehender Kompetenzen.

   Fragen:
   - Inwieweit hast du neue Kompetenzen erworben und welche sind das?
   - Inwieweit hast du bestehende Kompetenzen erweitern können und welche sind das?
   - Wie zufrieden bist du generell mit deinem Kompetenzerwerb?

4. Zusammenarbeit

   Erläuterung: Zusammenarbeit bezieht sich auf die Teamarbeit und die Kommunikation z.B. unter den Trainees oder den Mentor:innen, zwischen beiden Personengruppen, innerhalb der und zwischen den Institutionen.

   Fragen:
   - Wie hat die Kommunikation insgesamt funktioniert?
   - Wie hat die Teamarbeit insgesamt funktioniert?
   - Wie zufrieden bist du generell mit der Zusammenarbeit?

Onlinematerial 2. Evaluationsbogen

A: Fortbildungsinhalte

Wie bewerten Sie die Beiträge entsprechend folgender Skala in folgenden Themen?

Skala: stimme voll zu, stimme eher zu, stimme eher nicht zu, stimme nicht zu | keine Angabe

- *Umfang und Tiefe der Beiträge war angemessen.*
- *Die Beiträge waren gut strukturiert und verständlich.*
- *Die Inhalte und dazugehörigen Quellen waren aktuell.*
- *Die Beiträge deckten die relevanten Aspekte des Themas ab.*
- *Auf Fragen und Anregungen zum Thema wurde eingegangen.*
- *Die Inhalte der Beiträge waren neu für mich.*

Freitextfeld:

Platz für Kommentare und Anmerkungen, um einzelne Punkte zu erläutern

A1: ÖGD-Basics: Aufgaben und Tätigkeiten des Öffentlichen Gesundheitsdienstes
A2: ÖGD-Basics: „Wie funktioniert ein Gesundheitsamt?“ – Verwaltungs- und Entscheidungsstrukturen auf kommunaler Ebene
A3: Wissenschafts-Basics: ÖGD-Wissenschaft und Wissenschaft im ÖGD. Wo stehen wir?
A4: Wissenschafts-Basics: Erfolgreiche Publikationen planen. Wissenswertes zum wissenschaftlichen Publizieren
A5: Evidenzinformiertes Entscheiden und Handeln: Einführung in Evidenzbasierte Public Health
A6: Evidenzinformiertes Entscheiden und Handeln: Was heißt das für den kommunalen ÖGD?

Wie bewerten Sie die Beiträge entsprechend folgender Skala in folgenden Themen?

Skala: stimme voll zu, stimme eher zu, stimme eher nicht zu, stimme nicht zu | keine Angabe

- *Die Paneldiskussion war eine gute Ergänzung zu den anderen Beiträgen.*
- *Umfang und Tiefe der Diskussionsbeiträge waren angemessen.*
- *Die Beiträge der Panelist:innen waren gut strukturiert und verständlich.*
- *Es war möglich, Fragen zu stellen und das Thema zu diskutieren.*

Freitextfeld:

Platz für Kommentare und Anmerkungen, um einzelne Punkte zu erläutern

A7: Panel-Diskussion: Wie lassen sich wissenschaftliche Erkenntnisse schnell und effektiv in die tägliche Arbeit des ÖGD integrieren?

A: Moodlekurs

A8: Wie bewerten Sie den Moodlekurs „Wissenstransfer im ÖGD“ im Rahmen der ersten Blockveranstaltung?

Skala: stimme voll zu, stimme eher zu, stimme eher nicht zu, stimme nicht zu | keine Angabe

- *Umfang und Tiefe der zur Verfügung gestellten Materialien waren angemessen.*
- *Der Kurs ist gut strukturiert und verständlich aufgebaut.*
- *Die Inhalte und dazugehörigen Quellen waren aktuell.*
- *Die Inhalte decken die relevanten Aspekte des Themas ab.*
- *Die Möglichkeit, Fragen und Anregungen über das integrierte Forum zu notieren, finde ich gut.*

A8.1 Freitextfeld:

Platz für Kommentare und Anmerkungen, um einzelne Punkte zu erläutern

A8.2: Freitextfeld: Haben Sie Anregungen oder Wünsche für die Weiterentwicklung dieses Moodlekurses im Rahmen der Fortbildungsreihe „Wissenstransfer im ÖGD"?

A9: Propädeutikum „Der ÖGD im Nationalsozialismus" im Moodlekurs - Ich hatte die Möglichkeit, mich im Vorfeld mit dem online zur Verfügung gestellten Propädeutikum „Der ÖGD im Nationalsozialismus" auseinanderzusetzen.

- *Ja—> Weiterleitung zur nächsten Frage 9.1*
- *Nein – Weiterleitung zu 9.2*

9.1 Inwiefern stimmen Sie der nachfolgenden Aussage zu: Das Modul Propädeutikum „ÖGD im Nationalsozialismus" war eine gute Ergänzung zur Veranstaltung.

Skala: stimme voll zu, stimme eher zu, stimme eher nicht zu, stimme nicht zu | keine Angabe

9.2 Ich hatte bisher keine Möglichkeit, mich im Vorfeld mit dem online zur Verfügung gestellten Propädeutikum „Der ÖGD im Nationalsozialismus" auseinanderzusetzen, weil:

- *Ich bislang noch keine Zeit hatte, mich mit dem Propädeutikum auseinanderzusetzen.*
- *Ich kein Interesse am Thema habe.*
- *Ich keine Notwendigkeit sehe, mich mit dem Thema auseinanderzusetzen.*
- *mir die Inhalte des Themenfeldes bereits bekannt sind*
- *Sonstiges, und zwar:* _______________________

A10: Propädeutikum „Der ÖGD im Nationalsozialismus" im Moodlekurs - Sind Sie schon einmal mit dem Thema Medizinverbrechen im Nationalsozialismus in Berührung gekommen?

- *Ja -> Weiterleitung zu A10.1*
- *Nein*

A10.1 In welchem Kontext sind Sie mit dem Thema Medizinverbrechen im Nationalsozialismus in Berührung gekommen? Freitextnennung

A111: Sollte der ÖGD mehr dafür tun, um für das genannte Thema zu sensibilisieren? Freitextnennung

- *Ja -> Weiterleitung zu A11.1*
- *Nein*

A11.1 Was wäre dafür Ihrer Meinung nach der richtige Rahmen, um für das genannte Thema zu sensibilisieren?

B: Organisation und Durchführung

B1: Mit der Organisation der Veranstaltung war ich im Allgemeinen:

Skala: sehr zufrieden, eher zufrieden, eher nicht zufrieden, nicht zufrieden | keine Angabe

Freitextfeld:

Platz für Kommentare und Anmerkungen.

B2: Mit den folgenden Aspekten der Organisation und des Ablaufs der Veranstaltung war ich:

Skala: sehr zufrieden, eher zufrieden, eher nicht zufrieden, nicht zufrieden, keine Angabe

- Anmeldeverfahren und Vorabinformationen
- Teilnehmer:innen-Zahl
- Service und Betreuung während der Veranstaltung
- Moderation
- Zeitplan

Freitextfeld:

Platz für Kommentare und Anmerkungen, um einzelne Punkte zu erläutern

C: Gesamteindruck

C1: Wie bewerten Sie die Veranstaltung INSGESAMT?

Skala: stimme voll zu, stimme eher zu, stimme eher nicht zu, stimme nicht zu | keine Angabe

- *Der Veranstaltungsumfang inkl. Vor- und Nachbereitungszeit war angemessen.*
- *Die Auswahl der Beiträge deckte die relevanten Aspekte des Themas ab.*
- *Die vermittelten Inhalte waren gut aufeinander aufgebaut.*

Freitextfeld 1:

Besonders gut gefallen hat mir:

Freitextfeld 2:

Beim nächsten Mal könnte man Folgendes ändern oder verbessern:

C2: Meine Erwartungen an die Veranstaltung wurden erfüllt:

Skala: stimme voll zu, stimme eher zu, stimme eher nicht zu, stimme nicht zu | keine Angabe

Freitextfeld:

Platz für Kommentare und Anmerkungen zu Ihrer Einschätzung

C3: Haben Sie Anregungen oder Wünsche für die beiden Folgeveranstaltungen der Fortbildungsreihe „Wissenstransfer im ÖGD“?

Freitextfeld:

Ergänzende Fragen

D1: Ich bin auf diese Veranstaltung aufmerksam geworden durch:

- *Newsletter der AÖGW*
- *Social Media*
- *persönliche Empfehlung (z.B. durch Kolleg:innen)*
- *Verteiler meiner Hochschule*
- *sonstige Newsletter/Verteiler*
- *Fortbildungskalender der AÖGW*
- *Sonstiges und zwar:* ___________________________________

D2: Welche Aussage beschreibt Ihre aktuelle Tätigkeit am besten?

- *Ich arbeite aktuell im ÖGD auf kommunaler Ebene (z.B. in einem Gesundheitsamt).*
- *Ich arbeite aktuell im ÖGD auf Landesebene (z.B. in einem Landesgesundheitsamt).*
- *Ich arbeite aktuell im ÖGD auf Bundesebene (z.B. RKI).*
- *Ich studiere zurzeit.*
- *Ich arbeite aktuell an einer Hochschule (z.B. Universität oder Hochschule für Angewandte Wissenschaft).*
- *Ich arbeite aktuell bei einem privaten Unternehmen*
- *Keine der Aussagen trifft zu.*

Onlinematerial 3. Coding Frame

Tabelle 1. Inhaltsanalytische Auswertung der Gesprächsprotokolle (Verlaufsgespräche während der Rotation)

Code	Definition
Arbeitsergebnisse	Beispielhafte Nennungen von Arbeitsergebnissen während der Rotationsphase.
Begleitmodul (Seminarreihe)	Aussagen zur Umsetzung der Seminarreihe:<br>– Feedback zu einzelnen Beiträgen<br>– Feedback zur Einbindung in die Rotation<br>– Feedback zur Organisation und zum Ablauf der Seminarreihe
Zufriedenheit	Aussagen zur allgemeinen Zufriedenheit mit den Inhalten der Rotationsphasen.
Zusammenarbeit	Zusammenarbeit mit verschiedenen Berufsgruppen und Abteilungen.
Lernziele	– Aussagen zur Klarheit und Formulierung der Lernziele<br>– Aussagen zur Umsetzung / Umsetzbarkeit der Lernziele<br>– Aussagen zur Struktur / Logik der Lernziele
(Kommunikations-) Strukturen Forschungsprojekt	Aussagen zum Aufbau von (Kommunikations-)Strukturen für das Forschungsprojekt.
Hindernisse	Probleme und Hürden, die während des gesamten Trainee-Zeitraums aufgetreten sind.
Forschungsprojekt	– Aussagen zum Forschungsprojekt und den dazugehörigen Lernzielen<br>– Aussagen zur Einbindung des Forschungsprojekts in das Logbuch bzw. die Lernzielstruktur<br>– Aufzählungen inhaltlicher Aspekte des Forschungsprojekts
Ausstattung	Aussagen in Bezug auf ausreichende finanzielle Ressourcen, räumliche Ausstattung und technische Mittel.
Zeit für Lernziele	Aussagen dazu, ob die zur Verfügung stehende Zeit ausreichte, um die Lernziele zu erreichen.
Zeit für das Forschungsprojekt	Aussagen dazu, ob die Zeit ausreichte, um am Forschungsprojekt zu arbeiten.
Zeit für den Arbeitsalltag	Aussagen dazu, ob die Zeit ausreichte für institutionenspezifische Aufgaben im Arbeitsalltag.
Personal	– Aussagen zur Qualifikation des Personals für die Teilnahme<br>– Aussagen zu personellen Ressourcen
Verbesserungsvorschläge	Vorschläge von Mentor:innen und Trainees zu allen Aspekten des Rotationsprogramms.

Tabelle 2. Inhaltsanalytische Auswertung der Feedbackprotokolle (wöchentliche Kurzfeedbacks)

Code	Subcode	Definition
Forschungsprojekt	Fachliche Begleitung	Aussagen zur fachlichen Begleitung während des Forschungsprojekts:<br>– Nutzen der Rücksprachen mit Mentor:innen<br>– Umsetzung der Begleitung in den Institutionen<br>– Themen, die besprochen wurden
	Inhalt	Aussagen zur inhaltlichen Gestaltung des Projekts:<br>– Ausrichtung des Forschungsprojekts<br>– Umsetzung und Fortschritt<br>– Inhaltliche Diskussionspunkte
	Zeit	Aussagen zur zeitlichen Ausstattung für das Projekt: War die Zeit ausreichend im Hinblick auf die Projektziele?
Lernziele	Fachliche Begleitung	Aussagen zur fachlichen Unterstützung bei der Umsetzung der Lernziele:<br>– Nutzen von Rücksprachen mit Mentor:innen<br>– Umsetzung der Begleitung in den Institutionen<br>– Geführte Gespräche zur Umsetzung
	Formulierung	Aussagen zur Formulierung der Lernziele:<br>– Verständlichkeit<br>– Berücksichtigung der Umsetzungsebene
	Umsetzbarkeit	Aussagen zur Umsetzbarkeit der Lernziele: Inwieweit war die Umsetzung in den Institutionen erfolgreich?
	Zeit	Aussagen zur zeitlichen Ausstattung zur Umsetzung der Lernziele: War die Zeit ausreichend im Hinblick auf die Ziele?
Teamarbeit und Kommunikation	Trainees und Team	Aussagen zur Zusammenarbeit zwischen Trainees und Teams in den Partnerinstitutionen:<br>– Wie funktionierte die Zusammenarbeit?<br>– Welche Treffen fanden statt?<br>– Fühlten sich die Trainees wohl?
	Trainees und Mentor:innen	Aussagen zur Zusammenarbeit zwischen Trainees und Mentor:innen:<br>– Wie funktionierte die Zusammenarbeit?<br>– Welche Treffen oder Austausche fanden statt?<br>– Welche Herausforderungen gab es?
	Trainees untereinander	Aussagen zur Zusammenarbeit unter Trainees:<br>– Wie funktionierte die Zusammenarbeit?<br>– Wie verlief die Kommunikation?<br>– Welche Austauschformate gab es?<br>– Welche Herausforderungen traten auf?
Technik und Ausstattung	–	Aussagen zur technischen Ausstattung während der Rotation:<br>– Nutzung von Clouds/SharePoints<br>– Tools für Präsentationen und Online-Meetings<br>– Schnittstellenprobleme zwischen Institutionen<br>– verwendete Hardware
Hindernisse	–	Allgemeine Aussagen zu Hürden während der Rotation, z. B.:<br>– Urlaubs- und Abwesenheitszeiten<br>– Aufgabenverteilung<br>– Zeitliche Engpässe<br>– Vereinbarkeit von Rotation und Alltag

Onlinematerial 4. Persönliche Merkmale des Forschungsteams

Die folgende Tabelle enthält Angaben zu den persönlichen Merkmalen der Mitglieder des Forschungsteams, die an der Kodierung, Inhaltsanalyse und späteren Zusammenführung der Ergebnisse beteiligt waren. Diese Informationen werden gemäß den Anforderungen der COREQ-Checkliste (COnsolidated criteria for REporting Qualitative research) dargestellt, wobei insbesondere auf Bereich 1 der Checkliste eingegangen wird [31]. Bereich 1 betont die Bedeutung der Transparenz hinsichtlich der Merkmale des Forschungsteams, da diese die Interpretation und Analyse qualitativer Daten beeinflussen können.

Tabelle 3. Persönliche Merkmale der Mitglieder des Forschungsteams

	Qualifikationen	Beruf zum Zeitpunkt der Studie	Geschlecht	Für die Studie relevante methodische Erfahrung und Ausbildung
LA	PhD International Health an der Maastricht University;<br>MSc Epidemiologie;<br>MA Gesundheitsförderung;	Referentin für Epidemiologie und Gesundheitsberichterstattung und Leitung des Teams Angewandte ÖGD-Forschung und Transfer an der Akademie für Öffentliches Gesundheitswesen	w	Ausbildung in qualitativer Forschung an der Hochschule Ravensburg Weingarten (Deutschland) und der Ludwig-Maximilians-Universität (Deutschland); Leitung und Abschluss mehrerer Forschungsprojekte unter Verwendung qualitativer Verfahren
SB	MSc Governance und Leadership im europäischen Gesundheitswesen;<br>MA Gesundheitsversorgung, -ökonomie und -management	Wissenschaftlicher Mitarbeiter an der Akademie für Öffentliches Gesundheitswesen	m	Kurse und individuelle Fortbildungen in qualitativer Forschung an der Universität Bremen (Deutschland) und der Universität Maastricht (Niederlande); Durchführung, Auswertung und Analyse von Interviews im Rahmen von Forschungsprojekten
SG	PhD Medizinische Soziologie an der Heinrich-Heine-Universität	Wissenschaftlicher Mitarbeiter am Institut für Medizinische Soziologie der Heinrich-Heine-Universität / Universitätsklinikum Düsseldorf	m	Kurse in qualitativer Forschung an Qualitative Forschung an der Heinrich-Heine-Universität (Deutschland); Durchführung, Auswertung und Analyse von Interviews im Rahmen von Forschungsprojekten
HS	MSc Management und Qualitätsentwicklung im Gesundheitswesen	Referentin für Qualitätssicherung und Entwicklung an der Akademie für Öffentliches Gesundheitswesen	w	Kurse in qualitativer Methodik an der Alice Salomon Hochschule (Germany); Durchführung, Auswertung und Analyse von Interviews im Rahmen von Forschungsprojekten
SW	Promotion in Soziologie und Habilitation in Medizinischer Soziologie	Arbeitsgruppenleitung „Präventionsforschung" am Institut für Medizinische Soziologie der Heinrich-Heine-Universität / Universitätsklinikum Düsseldorf	w	Kurse in qualitativer Forschung an Qualitative Forschung an der Heinrich-Heine-Universität (Deutschland); Leitung und Abschluss mehrerer Forschungsprojekte unter Verwendung qualitativer Inhaltsanalyse

Onlinematerial 5. TIDieR checklist

Tabelle 4 Template for Intervention Description and Replication (TIDieR) checklist and guide [30]

Item	Where located	
	Primary paper	Other (details)
Brief name		
Provide the name or a phrase that describes the intervention.	Introduction	
Why		
Describe any rationale, theory, or goal of the elements essential to the intervention.		[24]
What		
Materials: Describe any physical or informational materials used in the intervention, including those provided to participants or used in intervention delivery or in training of intervention providers. Provide information on where the materials can be accessed (e.g., online appendix, URL).	Methods, Discussion *Fehler! Verweisquelle konnte nicht gefunden werden.*	[32, 45]
Procedures: Describe each of the procedures, activities, and/or processes used in the intervention, including any enabling or support activities		
Who Provided		
For each category of intervention provider (e.g., psychologist, nursing assistant), describe their expertise, background and any specific training given.		Supplement D
How		
Describe the modes of delivery (e.g., face-to-face or by some other mechanism, such as internet or telephone) of the intervention and whether it was provided individually or in a group.	Methods	[47]
Where		
Describe the type(s) of location(s) where the intervention occurred, including any necessary infrastructure or relevant features.	Introduction, Methods	
WHEN And HOW MUCH		
Describe the number of times the intervention was delivered and over what period of time including the number of sessions, their schedule, and their duration, intensity or dose.	Methods	
Tailoring		
If the intervention was planned to be personalized, titrated or adapted, then describe what, why, when, and how.		[38]
Modification		
If the intervention was modified during the course of the study, describe the changes (what, why, when, and how).		[48]
How well		
Planned: If intervention adherence or fidelity was assessed, describe how and by whom, and if any strategies were used to maintain or improve fidelity, describe them.	n.a.	
Actual: If intervention adherence or fidelity was assessed, describe the extent to which the intervention was delivered as planned.	n.a.	

Onlinematerial 6. COREQ Checklist

Tabelle 5. COnsolidated criteria for REporting Qualitative research (COREQ) checklist [31]

Topic	Item No.	Guide Questions/Description	Reported in section
**Domain 1: Research team and reflexivity**			
Personal characteristics			
Interviewer/facilitator	1	Which author/s conducted the interview or focus group?	Methods
Credentials	2	What were the researcher's credentials? (e.g., PhD, MD)	Supplement D
Occupation	3	What was their occupation at the time of the study?	
Gender	4	Was the researcher male or female?	
Experience and training	5	What experience or training did the researcher have?	
Relationship With Participants			
Relationship established	6	Was a relationship established prior to study commencement?	Methods
Participant knowledge of the interviewer	7	What did the participants know about the researcher? (e.g., personal goals, reasons for doing the research)	Methods
Interviewer characteristics	8	What characteristics were reported about the interviewer/facilitator? e.g., bias, assumptions, reasons and interests in the research topic	N.A.
**Domain 2: Study Design**			
Theoretical Framework			
Methodological orientation and Theory	9	What methodological orientation was stated to underpin the study? (e.g., grounded theory, discourse analysis, ethnography, phenomenology, content analysis)	Methods
Participant Selection			
Sampling	10	How were participants selected? (e.g., purposive, convenience, consecutive, snowball)	Methods
Method of approach	11	How were participants approached? (e.g., face-to-face, telephone, mail, email)	
Sample size	12	How many participants were in the study?	
Non-participation	13	How many people refused to participate or dropped out? Reasons?	
Setting			
Setting of data collection	14	Where was the data collected? (e.g., home, clinic, workplace)	Methods
Presence of non-participants	15	Was anyone else present besides the participants and researchers?	
Description of sample	16	What are the important characteristics of the sample? (e.g., demographic data, date)	Results
Data collection			
Interview guide	17	Were questions, prompts, guides provided by the authors? Was it pilot tested?	n.a.
Repeat interviews	18	Were repeat interviews carried out? If yes, how many?	n.a.
Audio/visual recording	19	Did the research use audio or visual recording to collect the data?	Methods
Field notes	20	Were field notes made during and/or after the interview / focus group / workshops?	Methods
Duration	21	What was the duration of the interviews / focus group / workshops?	Methods
Data saturation	22	Was data saturation discussed?	Results
Transcripts returned	23	Were transcripts / synthesized notes returned to participants for comment and/or correction?	n.a.
**Domain 3: analysis and findings**			
Data analysis			
Number of data coders	24	How many data coders coded the data?	Methods
Description of the coding tree	25	Did authors provide a description of the coding tree?	Supplement C
Derivation of themes	26	Were themes identified in advance or derived from the data?	Methods
Software	27	What software, if applicable, was used to manage the data?	Methods
Participant checking	28	Did participants provide feedback on the findings?	Methods
Reporting			
Quotations presented	29	Were participant quotations presented to illustrate the themes/findings? Was each quotation identified? (e.g., participant number)	not given
Data and findings consistent	30	Was there consistency between the data presented and the findings?	Results
Clarity of major themes	31	Were major themes clearly presented in the findings?	
Clarity of minor themes	32	Is there a description of diverse cases or discussion of minor themes?	
